# Supplementary material for: Ipragliflozin Ameliorates Diabetic Nephropathy Associated with Perirenal Adipose Expansion in Mice
Source: Int J Mol Sci. 2021 Jul 8;22(14):7329. doi: 10.3390/ijms22147329 (PMC8304702; doi:10.3390/ijms22147329)
Supplement: Supplementary file 1 [file ijms-22-07329-s001.zip › ijms-1253473-supplementary.pdf]

SUPPLEMENTARY MATERIALS AND METHODS

Biochemical assays

Blood glucose levels were measured using a glucometer (One-Touch Verio ® IQ; LifeScan Japan Co., Ltd., Tokyo, Japan). Serum total cholesterol, non-esterified fatty acid (NEFA), triglyceride (TG), and 3-hydroxybutyrate (3-HBA) concentrations were determined using the Wako Cholesterol E, NEFA C-Test Wako, TG E-Test Wako (Wako Pure Chemical Industries, Ltd., Osaka, Japan), and beta Hydroxybutyrate Assay Kit (Abcam plc, Cambridge, UK), respectively. Levels of insulin (Morinaga Institute of Biological Science, Inc., Kanagawa, Japan) and leptin (FUJIFILM Wako Shibayagi Co., Gunma, Japan) were measured using enzyme-linked immune sorbent assay kits.

Histological analysis

The kidney and PRAT were fixed with 4% paraformaldehyde, embedded in paraffin, and prepared as slides (4-μm sections). PRAT sections were stained with hematoxylin and eosin (HE), whereas kidney sections were stained with periodic acid-Schiff stain (PAS). To measure adipocyte cell size, >250 cells per section were counted using NIH Image J software. Macrophages in the kidney and PRAT were immunohistochemically measured using a rat monoclonal F4/80 antibody (MCA497GA; Abd Serotec, Kidlington, UK). Primary antibody binding was visualized using DAB + chromogen (Dako, Glostrup, Denmark). To evaluate fibrosis, the kidney and PRAT were stained using Sirius red, after which positive areas were measured using NIH Image J software. TUNEL staining of the PRAT was performed using the ApopTag Plus Peroxidase In Situ Apoptosis Detection Kit (Millipore, Billerica, MA, USA) according to the manufacturer's instructions. TUNEL-positive cells were then counted over the entire area of the section. All microscopic images were acquired using the Keyence BZ-9000 microscope.

Quantitative reverse transcription polymerase chain reaction

Total RNA of the kidney and PRAT was isolated using Sepasol reagent (Nacalai Tesque, Inc.). RNA was reverse-transcribed using Random Primer (Thermo Fisher Scientific Inc., Waltham, MA, USA) and ReverTra Ace (Toyobo Co., Ltd., Osaka, Japan). The quantitative reverse transcription polymerase chain reaction was performed using StepOnePlus Real-time PCR System with Fast SYBR Green Master Mix Reagent (Thermo Fisher Scientific Inc.). Primers are listed in the Supplementary Table. Data were normalized to the 36b4 levels and analyzed using the comparative CT method.

Western blotting

Kidney and PVAT were homogenized in lysis buffer (2% sodium dodecyl sulfate, 4 M urea, 1 mM ethylenediaminetetraacetic acid, 150 mM NaCl, 50 mM Tris, pH 8.0). Immunoblotting was performed using phospho-Akt (Ser473) antibody (9271, Cell Signaling Technology, Danvers, MA, USA), total Akt (9272, Cell Signaling Technology), perilipin-1 (3470, Cell Signaling Technology), HMGB1 (6893, Cell Signaling Technology), β-actin (4970, Cell Signaling Technology), phospho-p38 MAPK (Thr180/Tyr182) (4511, Cell Signaling Technology), p38 MAPK (8690, Cell Signaling Technology), and proliferating cell nuclear antigen (PCNA) (ab18197, Abcam, Cambridge, U.K.). Immunoblots were analyzed using ECL Prime Western Blotting Detection Reagent and ImageQuant LAS 4000 mini (GE Healthcare, Little Chalfont, UK).

Insulin signaling analysis

Mice were injected with 5 U/kg of human insulin (Humulin R, Eli Lilly and Company, Indianapolis, IN, USA) via the portal vein after 16 h of fasting. PRAT was then homogenized in lysis buffer, after which immunoblotting was performed using phospho-Akt (Ser473) antibody (9271, Cell Signaling Technology, Danvers, MA, USA) and total Akt (9272, Cell Signaling Technology).

Conditioned media collection from perirenal adipose tissue

Experimental mice (n = 6 in each group) were sacrificed using intraperitoneal pentobarbital anesthesia. Thereafter, PRAT was dissected in Dulbecco's Modified Eagle Medium with 1% penicillin/streptomycin (P/S) and 0.25% bovine serum albumin (BSA). Tissue samples from each mouse were weighed (100 mg), minced 20 times, and then incubated with 1 mL of Dulbecco's Modified Eagle Medium with 1% P/S and 0.25% BSA in 24-well culture plates (Costar, Corning, NY, USA) at 37 °C for 24 h. Conditioned media from PRAT (PRAT-CM) were centrifuged, frozen, and stored at −80 °C until use. Leptin levels in PRAT-CM were determined using the Mouse Leptin ELISA kit (FUJIFILM Wako Shibayagi Co., Gunma, Japan). Cells were incubated with PRAT-CM to evaluate the effects of PRAT-derived leptin on GEC proliferation.

Cell culture

Mouse GECs were obtained from Cell Biologics, Inc. (Chicago, USA). To determine the proliferative effects of PRAT-derived leptin on GECs and the protective effects of Ipra, GECs were cultured in PRAT-CM with or without leptin tA and the p38 MAPK inhibitor SB 203580. Thereafter, GECs were divided into seven groups: SD-CM (incubated with PRAT-CM from mouse fed SD for 48 h), HFD-CM (incubated with PRAT-CM from mouse fed HFD for 48 h), HFD-Ipra-CM (incubated with PRAT-CM from mouse fed HFD-Ipra for 48 h), HFD-CM-SB 203580 (pretreated with 10 μM SB203580 for 24 h and then incubated with PRAT-CM from mouse fed HFD for 48 h), HFD-CM-Leptin tA (pretreated with 10 μM leptin tA for 24 h and then incubated with PRAT-CM from mouse fed HFD for 48 h), HFD-Ipra-CM-SB 203580 (pretreated with 10 μM SB203580 for 24 h and then incubated with PRAT-CM from mouse fed HFD-Ipra for 48 h), and HFD-Ipra-CM-Leptin tA (pretreated with 10 μM leptin tA for 24 h and then incubated with PRAT-CM from mouse fed HFD-Ipra for 48 h). Further proof-of-principle studies were performed to determine the concentration-dependent response of GECs to leptin administration with or without the p38 MAPK inhibitor, the PI3K inhibitor, and leptin tA, as indicated in the Results section.

Isolation of adipocytes and stromal vascular fraction cells from perirenal adipose tissue

PRAT was weighed, minced, and digested in 15 mL of collagenase type 2 solution (2 mg/mL, Worthington) for 20 min at 37 °C with gentle shaking. The digestion mixture was centrifuged at 500 *g* for 3 min. Floating adipocytes were collected for RNA extraction, while pellets containing stromal vascular fraction (SVF) cells were suspended in phosphate-buffered saline. The suspension was passed through a 100-μm nylon mesh filter (BD Falcon) and centrifuged at 500 *g* for 3 min to pellet SVF cells.

Flow cytometry

Isolated SVF cells were resuspended in 200 μL phosphate-buffered saline containing 0.25% BSA, 0.2 mM ethylenediaminetetraacetic acid, and 1% penicillin/streptomycin. Cells were preincubated for 7 min at 4 °C in Fc Block (CD16/32, BD Biosciences) and then stained for 15 min with fluorophore-conjugated antibodies at 4 °C. The following antibodies were used: anti-CD45 (clone: 30-F11, BioLegend), anti-F4/80 (clone: BM8, BioLegend), anti-CD11b (clone: M1/70, BioLegend), anti-CD11c (clone: N418, BioLegend), and anti-CD206 (clone: C068C2, BioLegend). Flow cytometric analysis was performed using FACSCantoII (BD Biosciences), while cell sorting was performed using FACSAriaII (BD Biosciences). Data were analyzed using FlowJo software (v9.4.10, Tree Star).

Renal vein sampling

For renal vein sampling, animals were anesthetized, and the right kidney was exposed through a flank incision. After vein clamping, blood samples were collected using a 31-G needle.

Supplementary Table. List of primers for qPCR analysis (continued on the next page)

Gene Product		
Atgl	Fw	CCACTCACATCTACGGAGCC
	Rv	GATGCAGAGGACCCAGGAAC
Hsl	Fw	CGCTACACAAAGGCTGCTTC
	Rv	AACTGGCGGTCACACTGAG
Pck1	Fw	ATGAAAGGCCGCACCATGTA
	Rv	GGGCGAGTCTGTCAGTTCAA
Il-15	Fw	GTCTCCCTAAAACAGAGGCCA
	Rv	CAGTAACTTTGCAACTGGGATGA
Emr1	Fw	CTTTGGCTATGGGCTTCCAGTC
	Rv	GCAAGGAGGACAGAGTTTATCGT
Tnf	Fw	TCTACTGAACTTCGGGGTGA
	Rv	CTCCTCCACTTGGTGGTTTG
Adipoq	Fw	CTCCTGGAGAGAAGGGAGAG
	Rv	CCTTCAGCTCCTGTCATTCC
Col1a2	Fw	TTGCAATCGGGATCAGTACGA
	Rv	CACGTGGTCCTCTGTCTCCAG
Col6a3	Fw	CTGTCGCCTGCATTCATCC
	Rv	ACAACCCTCTGCACAAAGTC
Tgfb1	Fw	GTCACTGGAGTTGTACGGCA
	Rv	GGGCTGATCCCGTTGATTTC
Lep	Fw	TCCAGGATGACACCAAAACCC
	Rv	TGAAGTCCAAGCCAGTGACC
Angptl2	Fw	CACCTACAACCGCATCATCA
	Rv	TCCATGGACCTGATGGCTTA
Pdgfb	Fw	CTTCCTCTCTGCTGCTACCT
	Rv	AGCCCCATCTTCATCTACGG
Nampt	Fw	CAGTGGCCACAAATTCCAGA
	Rv	CCTATGCCAGCAGTCTCTTG
ObRa	Fw	GAAGTCTCTCATGACCACTACAGAT
	Rv	TTGTTTCCCTCCATCAAAATGTAA
ObRb	Fw	GCATGCAGAATCAGTGATATTTGG
	Rv	CAAGCTGTATCGACACTGATTTCTTC
Sglt2	Fw	TTGGTGTTGGCTTGTGGTCT
	Rv	ATGTTGCTGGCGAACAGAGA
Cd206	Fw	CTGAAAGGTGACCCTGGCAT
	Rv	GGTGGATTGTCTTGTGGAGC

Ccl2	Fw	CCACTCACCTGCTGCTACTCAT
	Rv	TGGTGATCCTCTTGTAGCTCTCC
Ccr2	Fw	ACAAATCAAAGGAAATGGAAGAC
	Rv	TGCCGTGGATGAACTGAGG
Il-6	Fw	CACTTCACAAGTCGGAGGCT
	Rv	CTGCAAGTGCATCATCGTTGT
Il-1b	Fw	CTCCAGCCAAGCTTCCTTGT
	Rv	TTGGAAGCAGCCCTTCATCT
Fabp4	Fw	AGACGACAGGAAGGTGAAGA
	Rv	TAACACATTCCACCACCAGC
Hmgb1	Fw	CCCGGATGCTTCTGTCAACT
	Rv	AGCAGACATGGTCTTCCACC
Cd36	Fw	TGTGGAGCAACTGGTGGATG
	Rv	CGTGGCCCGGTTCTAATTCA
Fabp1	Fw	GGGAAAAAGTCAAGGCAGTCG
	Rv	GACAATGTCGCCCAATGTCA
Srebf1	Fw	ATGCCATGGGCAAGTACACA
	Rv	ATAGCATCTCCTGCGCACTC
Fas	Fw	ATTTTGCTGTCAACCATGCC
	Rv	AGTTGAATCACTCCAACGGG
Ppara	Fw	GTGCAGCCTCAGCCAAGTT
	Rv	GTTGGATGGATGTGGCCAGG
Cpt1	Fw	TGGACCCAAATTGCAGTGGT
	Rv	GCATCTCCATGGCGTAGTAGT
36b4	Fw	GGCCCTGCACTCTCGCTTTC
	Rv	TGCCAGGACGCGCTTGT

Fw, forward primer; Rv, reverse primer.

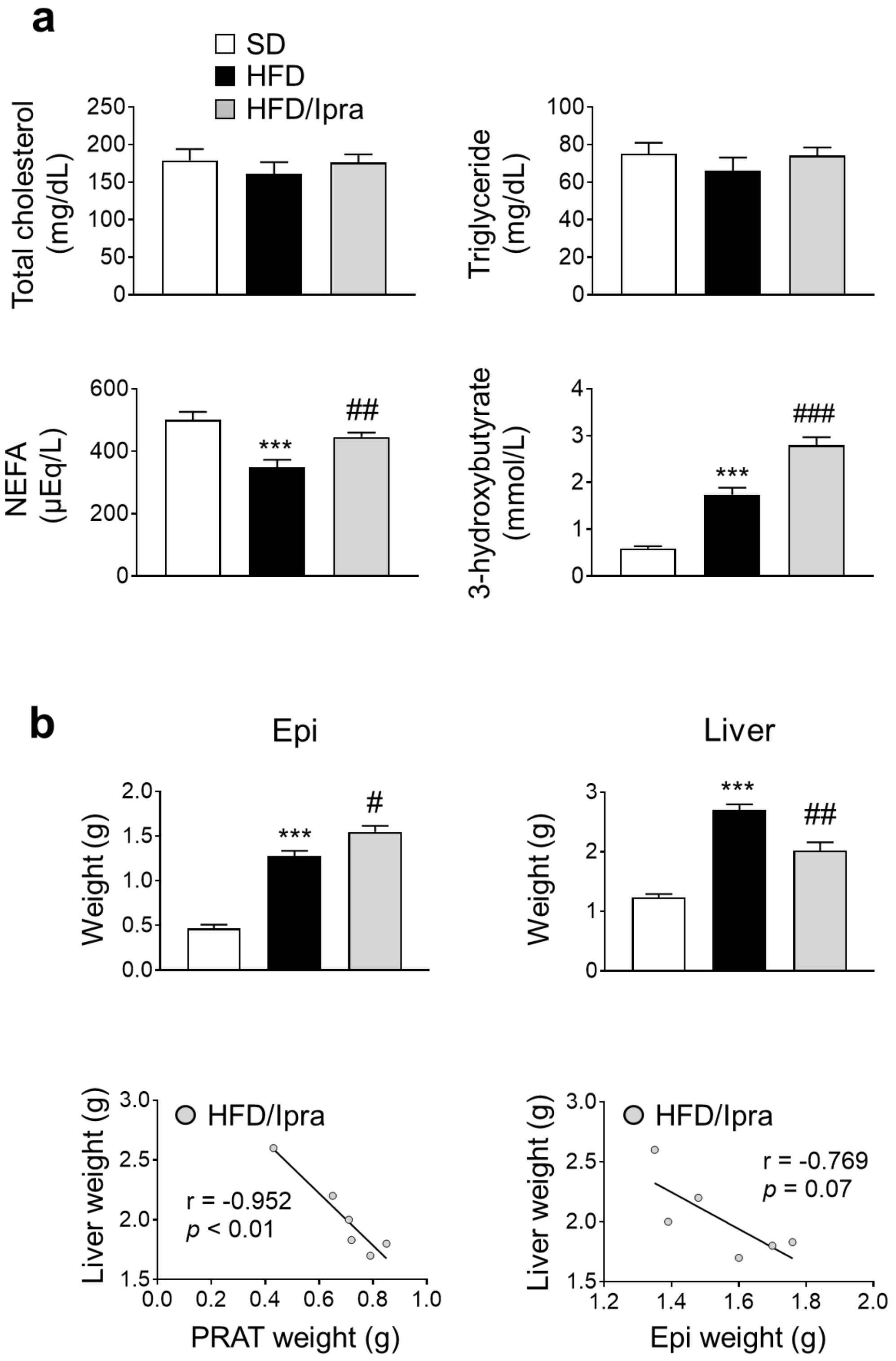

Supplementary Figure 1. Serum biochemical parameters and organ weights. (a) Serum lipid profile, 3-hydroxybutyrate concentration, and tissue weight of Epi and liver. (b) Correlation analyses between liver weight and each fat weight, PRAT, and Epi were conducted. Epi, epididymal adipose tissue; PRAT, perirenal adipose tissue. ***p < 0.001 vs. SD. #p < 0.05, ##p < 0.01, ###p < 0.001 vs. HFD. n = 9–11 (a) n = 6 (b).

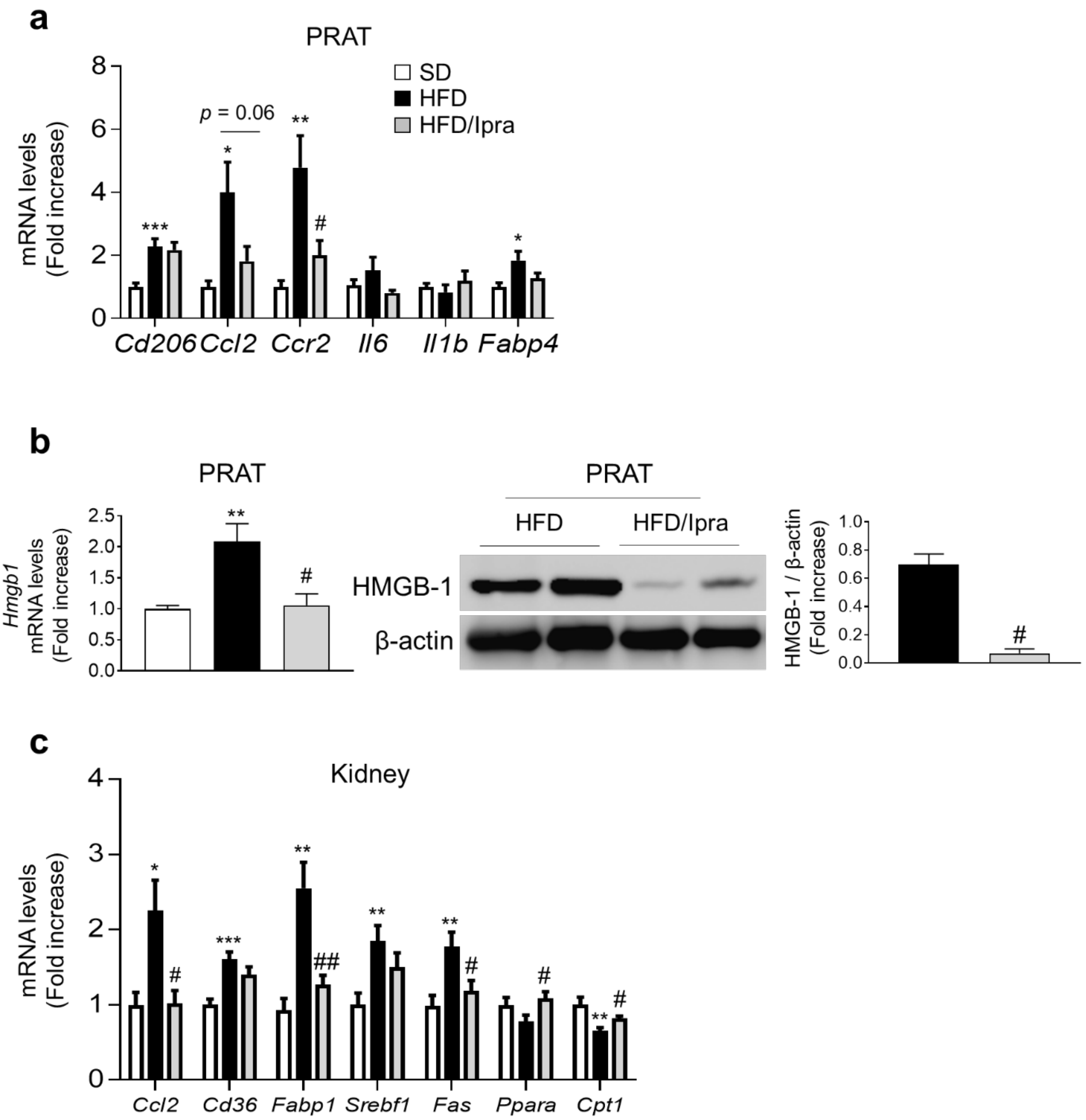

Supplementary Figure 2. Gene expression in PRAT and kidney. (a) Expression levels of inflammation-related genes in PRAT SD- or HFD-fed WT mice after 6 weeks of Ipra treatment. (b) Quantitative data of HMGB1 gene expression levels and protein levels in PRAT. (c) Expression levels of inflammation-, fatty acid transport-, fatty acid synthesis-, and lipolysis- related genes in kidney SD- or HFD-fed WT mice after 6 weeks of Ipra treatment. WT, wild-type; SD, standard diet; HFD, high-fat diet; Ipra, ipragliflozin; PRAT, perirenal adipose tissue. *p < 0.05, **p < 0.01, ***p < 0.001 vs. SD. #p < 0.05 vs. HFD. n = 6

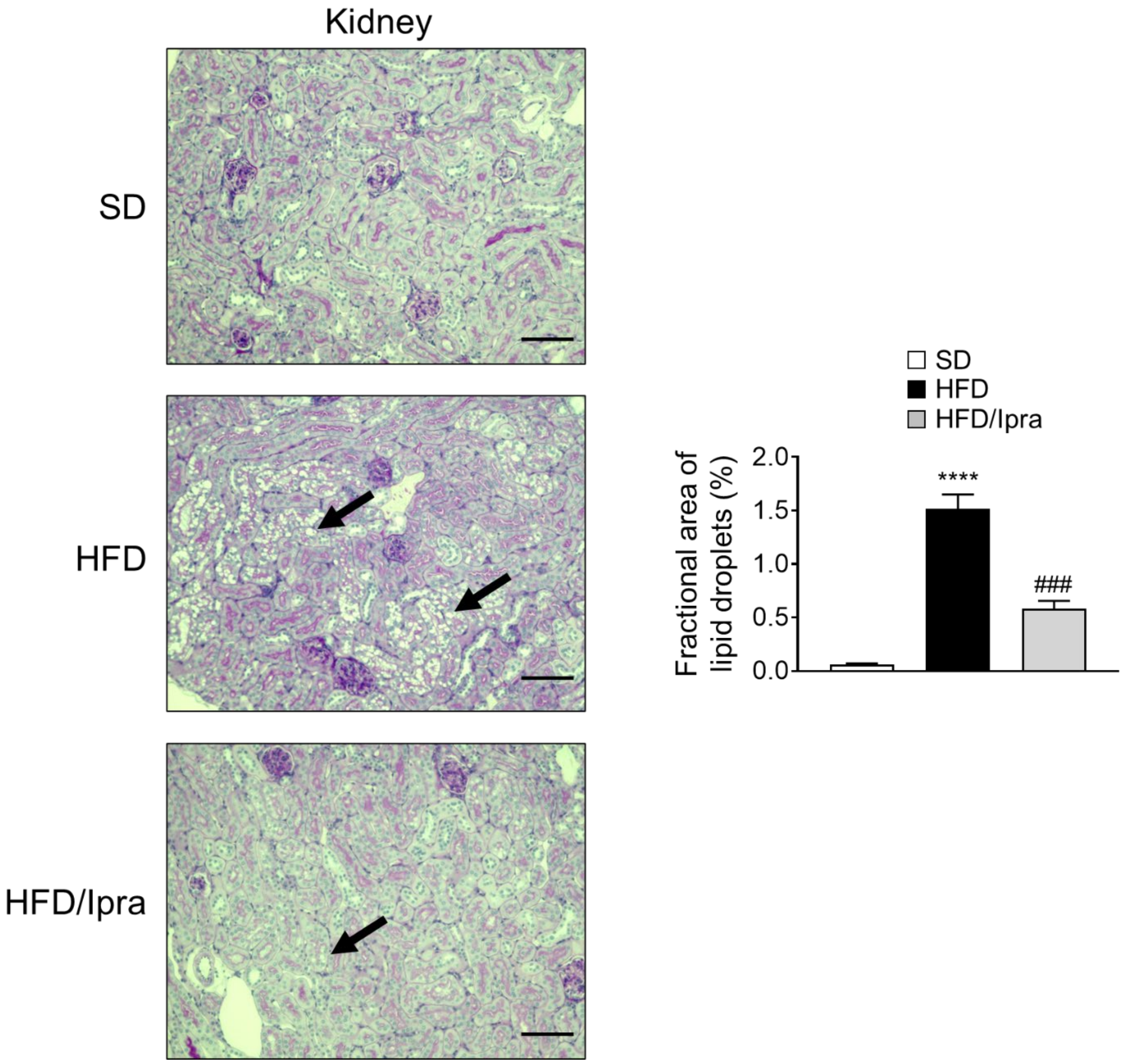

Supplementary Figure 3. Lipid accumulation in kidney. Representative images of PAS staining in kidney. Arrows show lipid droplets in renal tubular epithelial cells. Fractional area of lipid droplets was calculated as the ratio of the total amount of lipid droplets area to the whole tissue area. Scale bar, 150 μm. ***p < 0.001 vs. SD. ###p < 0.001 vs. HFD. n = 6

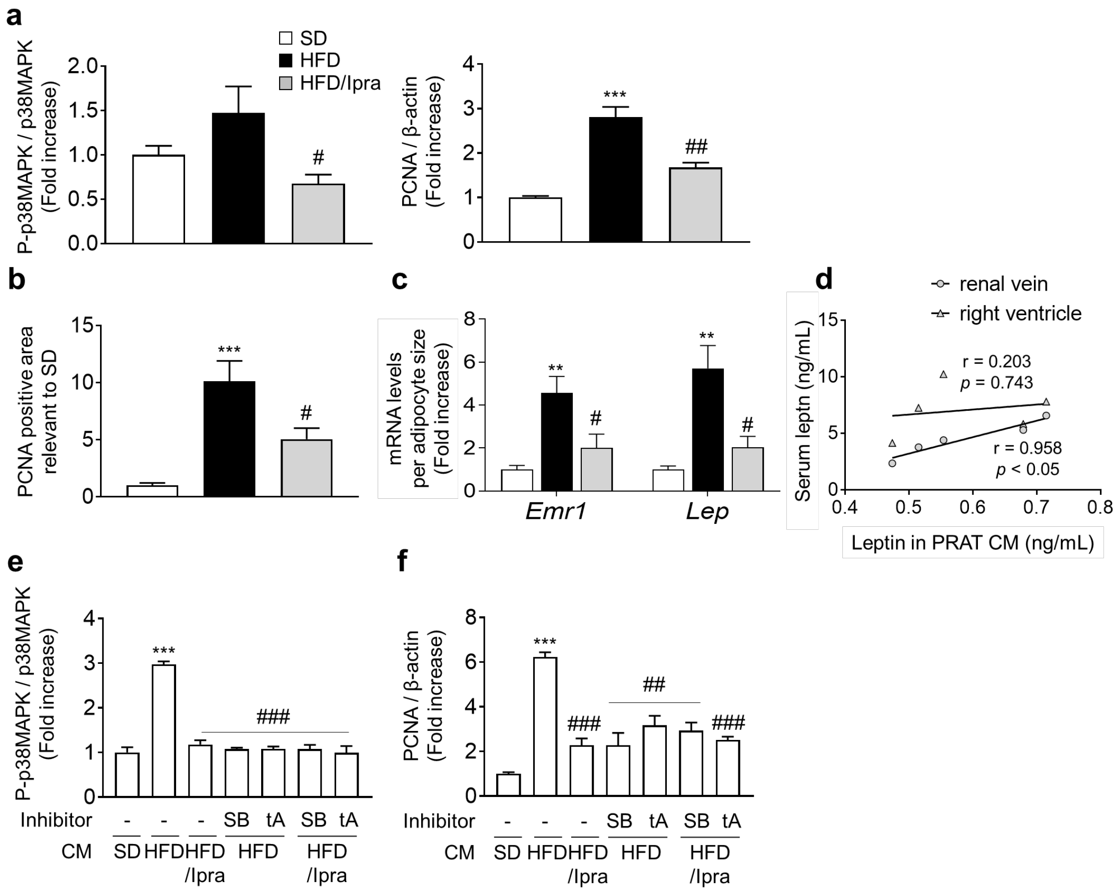

Supplementary Figure 4. (a) Quantitative data of phosphorylated/total p38 mitogen-activated protein kinase (p38 MAPK) and PCNA in the kidney. n = 6. (b) Quantitative data of fluorescent immunostaining of PCNA in the kidney. n = 6. (c) Gene expression levels of Emr1 and Lep per adipocyte size in PRAT SD- or HFD-fed WT mice after 6 weeks of Ipra treatment. n = 6 (d) Correlation analysis between leptin concentration in PRAT-CM and serum leptin concentration (in renal vein and right ventricle) in Ipra-treated mice. Quantitative data of (e) phosphorylated/total p38 MAPK and (f) PCNA/β-actin in mouse GECs treated with PRAT-CM after pre-incubation with SB and tA. **p < 0.01, ***p < 0.001 vs. SD. #p < 0.05, ##p < 0.01, ###p < 0.001 vs. HFD. n = 3.
